# Assessment of health inequality between urban-to-urban and rural-to-urban migrant older adults in China: a cross-sectional study

**DOI:** 10.1186/s12889-020-8341-5

**Published:** 2020-02-24

**Authors:** Sha Ma, Qiuju Li, Xudong Zhou, Weiming Cao, Minmin Jiang, Lu Li

**Affiliations:** 1 School of Public Health, The Institute of Social and Family Medicine, Zhejiang University, Xihu District, Hangzhou, Zhejiang Province 310058 People’s Republic of China; 2 School of Humanities and Social Sciences, Zhejiang Chinese Medical University, Gaoke Road, Fuyang District, Zhejiang Province 311402 People’s Republic of China

**Keywords:** Migrant older adults, Physical health, Mental health, Cognitive social capital, Social integration

## Abstract

**Background:**

Many studies focused on health inequality between migrant older adults and local older adults, while few study concerned the health inequalities between urban-to-urban and rural-to-urban migrant older adults. This study aimed to compare physical health and mental health between these two groups in Hangzhou, Zhejiang Province, China, and to explore the relationship between cognitive social capital, social integration and health among migrant older adults.

**Methods:**

A two-stage stratified sampling method was employed to recruit participants from May to August 2013 in Hangzhou. Measurement data were compared with student’s *t-*tests and multivariate analysis of variance (MANOVA). Multiple linear regression was adopted in this study.

**Results:**

A total of 1000 of participants who met the inclusion criteria were analyzed, consisting of 527 (52.7%) urban-to-urban and 473 (47.3%) rural-to-urban migrant older adults. There were no statistically significant difference in physical health and mental health between urban-to-urban and rural-to-urban groups on the whole. However, urban-to-urban migrant older adults had a higher reciprocity and social integration than did in rural-to-urban group (13.36 vs. 12.50, *p* < 0.01; 40.07 vs. 38.50, *p* < 0.01). And both of cognitive social capital and social integration were positively related to physical health (social reciprocity: t = 6.69, *p* < 0.01; social trust: t = 3.27, *p* < 0.01; social integration: t = 5.66, *p* < 0.01) and mental health (social reciprocity: t = 4.49, *p* < 0.01; social trust: t = 5.15, *p* < 0.01; social integration: t = 10.02, *p* < 0.01). Overall, the female, widowed, and the oldest among migrant older adults had a worse health.

**Conclusions:**

Social capital and social integration were played important roles in health of migrant older adults. The female rural-to-urban migrant older adults, those aged over 70 years, and older adults who were not in marriage should be especially concerned in health policy making.

## Background

The proportion of older adults (age 60 years and above) in the whole population was increasing worldwide [1], both in developed and developing countries [2]. In 2010 the Sixth National Population Census showed that people aged 60 years and above accounted for 13.6% of the total population in China [3]. In 2017 China migrant population development report announced that there were 24.5 million migrant [4]. The scale of migrant older adults, aged 60 and above who migrate between regions, increased rapidly. Migrant older adults’ health has been a big issue for society as that will become an important ingredient of health for all.

With medical model transformation from a biomedical model to a bio-psycho-social model, recent evidence has been show that factors such as social capital [5], social integration [6] and social-economic factors (gender, education, marital status, immigrant status) may be important for migrant older adults’ health. Among them, social capital and social integration were popular in international health research [7] and social science disciplines as well as the field of public health [8, 9]. Nan Lin [10] and other researchers [11, 12] defined social capital from different perspectives. Social capital refers to those features of social relationships--such as levels of interpersonal trust and norms of reciprocity and mutual aid--that facilitate collective action for mutual benefit [13]. In other words, social capital was defined as the resources available to individuals and groups through social connections and social relations with others [14]. Generally, social capital was divided into cognitive social capital and structural social capital, and horizontal social capital and vertical social capital [15]. Social integration was a broad term that refers to the degree to which an individual is connected to others and embedded in the community [16].

Previous studies suggested that poor social capital or social integration increases risk for poor health, the majority of studies focused on the association between social capital and physical health (or mental health) [17, 18] and the health differences between migrant older adults and local residents, but few studies explored health inequality between urban-to-urban and rural-to-urban migrant older adults, and the relationships between social integration and health [19], so little is been known about what health inequalities in these two groups. In this study, urban-to-urban migrant older adults refers to those aged 60 years and above who flowed from other cities to inflow areas (Hangzhou) above 6 months as temporary residents with non-agricultural household registration rather than permanent residents; rural-to-urban older adults refers to those aged 60 years and above who flowed from counties to inflow areas (Hangzhou) above 6 months as a temporary resident with agricultural household registration rather than permanent residents. Therefore, this study aimed to assess the differences of physical health and mental health between urban-to-urban and rural-to-urban migrant older adults, and to explore the relationship between social capital and social integration and physical health and mental health among urban-to-urban and rural-to-urban migrant older adults. Actually, social capital of migrant older adults might reduce when they left their birthplace to an unfamiliar city, which could influence their mental health and physical health. Furthermore, health advantage for rural-to-urban migrant older adults might worse than urban-to-urban migrant older adults because it was harder for those from rural area than others from urban area to adapt to an unfamiliar city life. Hypotheses are given as following. First, the rural-to-urban migrant older adults had a worse physical health than urban-to-urban group. Second, the rural-to-urban migrant older adults had a worse mental health than urban-to-urban one. Third, social capital and social integration played a crucial role in physical health and mental health among urban-to-urban and rural-to-urban migrant older adults.

## Methods

### Study design

Hangzhou is a well-developed city in China, which had a population of 7 million in 2012, per capital and total gross domestic products of 88,985 CNY (12,940.67 USD) and 780,398 billion CNY (113,489.58 USD), respectively [20], with a migrant worker population of 2.44 million, accounting for 57.5% of the province’s total population [21]. A population-based cross-sectional survey was conducted from May to August 2013 in Hangzhou, Zhejiang Province, China. Two-stage stratified sampling method was employed. First, Xihu District and Binjiang District, according to their economic status and the scale of migrant population, were selected as the study site from 13 districts in Hangzhou. Then, one sub-district was randomly selected from these two districts respectively, they were Sandun sub-district in Xihu District and Puyan sub-district in Binjiang District. Finally, two communities, Lilan and Houchengqiao in Sandun and Zhijiang and Lianzhuang in Puyan, were randomly recruited from each sub-district. All older adults met the inclusion criteria were recruited from these four communities.

### Participants

Participants were consisting of urban-to-urban and rural-to-urban migrant older adults. Urban-to-urban migrant older adults refers to those aged 60 years and above who flowed from other cities to inflow areas (Hangzhou) above 6 months as temporary residents with non-agricultural household registration rather than permanent residents. Rural-to-urban older adults refers to those aged 60 years and above who flowed from counties to inflow areas (Hangzhou) above 6 months as a temporary resident with agricultural household registration rather than permanent residents.

**Fig. 1:**
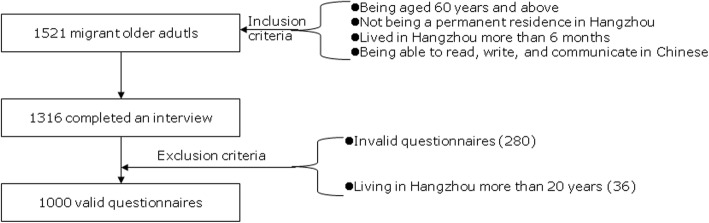
Flow chart on the process of participants selection

Recruitment process was shown in Fig. 1. All participants should met the following inclusion criteria: i) being aged 60 years old and above; ii) not being a registered and permanent residence in Hangzhou; iii) having lived in Hangzhou more than 6 months; and iv) being able to read, write, and communicate in Chinese, and not having a cognitive disorder. Exclusion criteria were: i) having not finish a half of a questionnaire; ii) illogical questionnaire (a questionnaire that participants answered inconsistent on particular questions); iii) being lived in Hangzhou more than 20 years. A total of 1521 participants met these inclusion criteria and enrolled, 1316 of them completed a face-to-face interview. A final total of 1000 questionnaires were deemed valid after performing a quality check. Thus, the response rate was 86.5% and rate of valid questionnaires was 76.0%.

### Ethical approval

Informed consent was obtained from participants in the form of verbal agreement and ethical approval for the study has been proved by Zhejiang University Ethical Committee (NO. ZGL201608–1).

### Measures

#### Socio-demographic characteristics

Socio-demographic characteristic included gender (male, female), age (60 to 69 years old, 70 years old or above), marital status (in marriage, not-in-marriage), educational attainment (primary school or below, junior high school or above), mainly economic source (oneself or spouse, offspring or others), years living in local city (6 months to 1 year, one to three years, three to six years, and six to twenty years) and weight status (low weight: BMI < 18.5; normal: 24 > BMI ≥ 18.5; overweight: 28 > BMI ≥ 24; and obesity: BMI ≥ 28). Weight status was rated by body mass index (BMI, a participant’s bodyweight in kilograms divided by body height in squared meters).

#### Cognitive social capital

“Social capital refers to a sense of community embeddedness, which is in part reflected by group membership, civic participation, and perceptions of trust, cohesion, and engagement” [22]. Cognitive social capital as a proxy of social capital has been widely accepted in the literature [23]. Social trust and reciprocity were used as a proxy for cognitive social capital [16]. The Cronbach’s Alpha of social trust was 0.77. Kaiser-Meyer-Olkin (K-M-O) was 0.83 (*p* < 0.01). The Cronbach’s Alpha of reciprocity was 0.8. Kaiser-Meyer-Olkin (K-M-O) was 0.77 (*p* < 0.01). The scale for measuring social capital and social trust had a good reliability and validity. All items in the Cognitive Social Capital Scale were valid. A 5-point Likert scale was used to measure the degree of agreement to each item of social trust and reciprocity. The responses were collapsed into 5-point scale: 1 = strongly disagree, 2 = disagree, 3 = neutrality, 4 = agree, and 5 = strongly agree. Higher total scores indicated higher social trust and reciprocity.

#### Social integration

“Social integration refers to the process in which individuals come together as a whole in a community through assimilation” [24], which can be seen as a dynamic and structured process in which all members communicate well with each other to achieve and maintain peaceful social relations. We adopted 10 items to reflect social integration of migrant older adults in this study. A 5-point Likert Scale was used to measure the degree of agreement in each item. Responses were collapsed into dichotomous outcomes: 1 = strongly disagree, 2 = disagree, 3 = neutrality, 4 = agree, and 5 = strongly agree. Higher total scores indicated higher social integration. The Cronbach’s Alpha of social integration was 0.62. Kaiser-Meyer-Olkin (K-M-O) was 0.80 (*p* < 0.01). Unless a item v) *my family members always quarrel with me for future living arrangements*, the remaining items in the Social Integration Scale were valid.

#### Physical health and mental health

Chinese version 36-item Short Form Health Survey (SF-36) was used as a comprehensive proxy of health. Calculation of SF-36 can be acquired in published literature [25]. This scale consists of eight components, including Physical Function (PF), Role Physical (RP), Bodily Pain (BP), General Health (GH), these four components constitute Physical Health (PH); the remaining are Vitality (VT), Social Function (SF), Role Emotional (RE), and Mental Health (MH), these four components constitute Mental Health (MH). Higher SF-36 score indicates a better health.

### Statistical analysis

Physical health and mental health were dependent variables, category of migrant older adults be designed as the independent variable. Gender, age, marital status, educational attainment, mainly economic source, years living in local city, weight status, cognitive social capital, and social integration were analyzed in this study as the covariates. Frequencies were calculated to describe the participants’ socio-demographic characteristics. Exploratory factor analysis was adopted to measure the validity of scales. Multivariate analysis of variance (MANOVA) was used to evaluate differences in physical health and mental health between urban-to-urban and rural-to-urban migrant older adults. Student’s *t-*tests and Univariate Analysis of Variance (ANOVA) were employed to evaluate difference in physical health and mental health by gender, age category, mainly economic source, marital status, educational attainment, years living in local city, and weight status. Multiple linear regression was adopted to explore relationships between migrant older adults and health (physical health and mental health) after controlling socio-demographic characteristics. Statistical analyses were conducted using SPSS version 18.0 for Windows.

## Results

### Socio-demographic characteristics

A total of 1000 migrant older adults finished the survey effectively, consisting of 527 (52.70%) urban-to-urban and 473 (47.30%) rural-to-urban migrant older adults. Overall, the number of female migrant older adults was 549 (54.90%), which was higher than the male 451 (45.10%) (*p* < 0.01) (Table 1). The majority of them aged 60 to 69 years both in urban-to-urban (411, 77.99%) and rural-to-urban (413, 87.32%) groups (*p* < 0.01). Proportion of the married migrant older adults was close in these two groups, 84.06% in urban-to-urban and 81.61% in rural-to-urban group respectively (*p* = 0.3). In terms of education, 32.14% of rural-to-urban migrant older adults had an education level of junior school or above, far below 76.85% of that in urban-to-urban migrant older adults (*p* < 0.01). The overwhelming majority (85.58%) of urban-to-urban migrant older adults were self-supporting or dependent on their spouse, compared with only 51.59% of rural-to-urban migrant older adults (*p* < 0.01). More than 60% urban-to-urban and rural-to-urban migrant older adults have lived in Hangzhou more than 3 years (*p* < 0.01). The weight status distribution between urban-to-urban and rural-to-urban groups was similar (37.76% of overweight in urban-to-urban and 30.49% in rural-to-urban migrant groups).

**Table 1:** Summary of the socio-demographic characteristic between urban-to-urban and rural-to-urban migrant old adults (*N* = 1000)

Variables	Urban-to-urban		Rural-to-urban		Total		*χ*^*2*^	*p*
	n	%	n	%	n	%		
Gender							8.01	< 0.01
Male	260	49.34	191	40.38	451	45.10		
Female	267	50.66	282	59.62	549	54.90		
Age							14.95	< 0.01
60 to 69 years	411	77.99	413	87.32	824	82.40		
70 years or above	116	22.01	60	12.68	176	17.60		
Marital status							1.06	0.3
In marriage	443	84.06	386	81.61	829	82.90		
Not-in-marriage	84	15.94	87	18.39	171	17.10		
Educational attainment							201.98	< 0.01
Primary school and be low	122	23.15	321	67.86	443	44.30		
Junior school and above	405	76.85	152	32.14	557	55.70		
Mainly economic source							135.88	< 0.01
Oneself or spouse	451	85.58	244	51.59	695	69.50		
Offspring or others	76	14.42	229	48.41	305	30.50		
Years living in local city							15.74	< 0.01
6 months to 1 year	35	6.64	62	13.11	97	9.70		
1 to 3 years	132	25.05	115	24.31	247	24.70		
3 to 6 years	187	35.48	176	37.21	363	36.30		
6 to 20 years	173	32.83	120	25.37	293	29.30		
Weight status							9.22	0.03
Low weight	14	2.66	23	4.90	37	3.71		
Normal	291	55.22	274	58.42	565	56.73		
Overweight	199	37.76	143	30.49	342	34.34		
Obesity	23	4.36	29	6.18	52	5.22		

No difference of social trust score was obtained between urban-to-urban and rural-to-urban groups (21.38 vs. 21.43, *p* = 0.85), while urban-to-urban migrant older adults had higher score of reciprocity and score of social integration than that of rural-to-urban migrant older adults respectively (13.36 vs. 12.50, *p* < 0.01; 40.07 vs. 38.5, *p* < 0.01) (Table 2).

**Table 2:** Difference of cognitive social capital and social integration between urban-to-urban and rural-to-urban migrant older adults

Items	Urban-to-urban		Rural-to-urban		t	*p*	95% CI for the difference	
	*n*	Mean	n	Mean			Lower	Upper
Score of social trust	448	21.38	361	21.43	−0.20	0.85	−0.61	0.50
Score of reciprocity	527	13.36	473	12.50	3.37	< 0.01	0.36	1.36
Score of social integration	515	40.07	461	38.50	3.95	< 0.01	0.79	2.35

### Comparison of physical health between urban-to-urban and rural-to-urban migrant older adults

Overall, few urban-to-urban and rural-to-urban migrant older adults reported a poor health in survey (3.61% vs. 5.92%). The urban-to-urban group had a higher physical health score than did the rural-to-urban group (309.72 vs. 299.3, *p* = 0.03). Specifically, the average score of Role Physical (RP) and Bodily Pain (BP) in urban-to-urban group were higher than that in rural-to-urban group respectively (77.47 vs. 72.67, *p* = 0.05; 82.56 vs. 78.32, *p* < 0.01) (Table 3).

**Table 3:** Difference of cognitive physical health and mental health between urban-to-urban and rural-to-urban migrant older adults

Items	Urban-to-urban		Rural-to-urban		t	*p*	95% CI for the difference	
	*n*	Mean	*n*	Mean			Lower	Upper
Total score of Physical Health	527	309.72	471	299.30	2.17	0.03	0.98	19.86
Physical Function (PF)	527	89.12	471	89.35	−0.23	0.82	−2.26	1.79
Role Physical (RP)	527	77.47	473	72.67	1.99	0.05	0.07	9.51
Bodily Pain (BP)	527	82.56	473	78.32	3.06	< 0.01	1.52	6.97
General Health (GH)	527	60.57	473	59.05	1.09	0.28	−1.23	4.27
Total score of Mental Health	512	306.88	461	299.09	1.74	0.08	−0.97	16.55
Vitality (VT)	527	64.79	473	63.78	0.83	0.41	−1.37	3.38
Social Function (SF)	513	85.82	462	82.49	2.93	< 0.01	1.09	5.55
Role Emotional (RE)	527	82.29	473	79.92	1.09	0.27	−1.89	6.64
Mental Health (MH)	526	75.05	472	74.35	0.65	0.51	−1.41	2.81

The urban-to-urban group had a higher physical score in two age groups than that in rural-to-urban group (315.85 vs. 305.60, *p* = 0.05; 287.98 vs. 256.12, *p* = 0.02) (Table 4). The married urban-to-urban migrant older adults had a higher physical health score than did those in rural-to-urban group (317.46 vs. 303.91, *p* = 0.01), while there were no difference between these two groups for those who were not in marriage. No differences were obtained between urban-to-urban and rural-to-urban groups for those with different levels of educational attainment (298.06 vs. 292.61, *p* = 0.50 for those with primary school and below; 313.23 vs. 313.46, *p* = 0.97 for those with junior high school and above). Self-supporting older adults had a similar physical health in urban-to-urban and rural-to-urban group (312.01 vs. 301.65, *p* = 0.09), so did for those depending on offspring or others (296.13 vs. 296.79, *p* = 0.95). Only for urban-to-urban migrant older adults lived in Hangzhou three to 6 years had a better physical health than that in rural-to-urban group (319.07 vs. 293.26, *p* < 0.01), the remaining had no difference in physical health between these two groups. Older adults with normal weight in urban-to-urban group had a higher physical health score than that in rural-to-urban group (312.56 vs. 287.56, *p* < 0.01).

**Table 4:** The differences in Physical Health score between urban-to-urban and rural-to-urban migrant old adults across socio-demographic characteristics

Variables	Urban-to-urban		Rural-to-urban		t (F)	*p*
	*n*	Mean	*n*	Mean		
Gender						
Male	260	310.90	191	301.04	1.33	0.18
Female	267	308.57	280	298.11	1.64	0.10
Age						
60 to 69 years	411	315.85	411	305.60	2.01	0.05
70 years or above	116	287.98	60	256.12	2.40	0.02
Marital status						
In marriage	443	317.46	384	303.91	2.66	0.01
Not-in-marriage	84	268.88	87	278.93	−0.80	0.43
Educational attainment						
Primary school and below	122	298.06	320	292.61	0.68	0.50
Junior school and above	405	313.23	151	313.46	−0.04	0.97
Mainly economic source						
Oneself or spouse	451	312.01	243	301.65	1.69	0.09
Offspring or others	76	296.13	228	296.79	−0.06	0.95
Years living in local city						
6 months to 1 year	35	309.75	61	317.10	0.23	0.63
One to Three years	132	313.81	115	297.47	3.01	0.08
Three to Six years	187	319.07	176	293.26	11.12	< 0.01
Six to Twenty years	173	296.48	119	300.87	0.22	0.64
Weight status						
Low weight	14	313.93	23	288.53	1.16	0.29
Normal	291	312.56	273	287.56	14.99	< 0.01
Overweight	199	305.93	142	321.64	3.87	0.05
Obesity	23	304.01	29	314.64	0.30	0.59

### Comparison of mental health score between urban-to-urban and rural-to-urban migrant older adults

No difference of mental health was obtained between urban-to-urban and rural-to-urban groups (306.88 vs. 299.09, *p* = 0.08). Specifically, urban-to-urban group had a higher Social Function (SF) score than that in rural-to-urban group (85.82 vs. 82.49, *p* < 0.01) (Table 3).

Participants aged 60 to 69 years had a higher mental health score in urban-to-urban group than that in rural-to-urban group (314.73 vs. 303.83, *p* = 0.02), while no difference was found in those aged 70 years or above (279.49 vs. 267.42, *p* = 0.32) (Table 5). The married had a higher mental health score in urban-to-urban group than did in rural-to-urban group (313.99 vs. 301.29, *p* = 0.01). Self-supporting older adults had a higher mental health in urban-to-urban group than did those in rural-to-urban group (307.23 vs. 291.22, *p* = 0.01). Participants lived in Hangzhou one to 3 years had a higher mental health score in urban-to-urban group than that in rural-to-urban group (312.19 vs. 288.95, *p* = 0.02). Older adults with normal weight had a higher mental health score in urban-to-urban group than that in rural-to-urban group (308.37 vs. 288.14, *p* < 0.01). No difference of mental health score was observed between urban-to-urban and rural-to-urban groups for the male (306.82 vs. 302.61, *p* = 0.52), so did for the female (306.94 vs. 296.67, *p* = 0.09). And no difference of mental health was detected between urban-to-urban and rural-to-urban groups across educational attainment.

**Table 5:** The differences in Mental Health score between urban-to-urban and rural-to-urban migrant old adults across socio-demographic characteristics

Variables	Urban-to-urban		Rural-to-urban		t (F)	*p*
	n	Mean	n	Mean		
Gender						
Male	255	306.82	188	302.61	0.64	0.52
Female	257	306.94	273	296.67	1.70	0.09
Age						
60 to 69 years	398	314.73	401	303.83	2.30	0.02
70 years or above	114	279.49	60	267.42	1.00	0.32
Marital status						
In marriage	429	313.99	374	301.29	2.73	0.01
Not-in-marriage	83	270.15	87	289.62	−1.55	0.12
Educational attainment						
Primary school and below	117	283.61	310	292.72	− 1.10	0.27
Junior school and above	395	313.77	151	312.18	0.27	0.79
Mainly economic source						
Oneself or spouse	437	307.23	237	291.22	2.61	0.01
Offspring or others	75	304.88	224	307.42	−0.30	0.76
Years living in local city						
6 months to 1 year	35	311.04	58	313.72	0.04	0.84
One to Three years	126	312.19	112	288.95	5.87	0.02
Three to Six years	180	312.61	172	298.84	3.77	0.05
Six to Twenty years	171	296.08	119	301.88	0.49	0.49
Weight status						
Low weight	14	315.88	22	289.52	1.76	0.19
Normal	281	308.37	269	288.14	10.70	< 0.01
Overweight	194	303.08	141	317.55	4.26	0.04
Obesity	23	315.31	25	318.30	0.02	0.88

### Influencing factors on physical health and mental health based on multiple linear regression

As Table 6 showed, urban-to-urban migrant older adults had a higher physical health score than that in rural-to-urban migrant older adults (Model PH1), as well as mental health score was higher in urban-to-urban group than that in rural-to-urban group (Model MH1). However, these differences were disappeared after controlling for socio-demographic factors (*Model PH2, Model MH2, Model PH3, Model MH3*). For those with a higher cognitive social capital and social integration had a better physical health and mental health than those migrant older adults with a lower cognitive social capital and social integration. With increasing of score of social reciprocity, migrant older adults showed a better physical health (*t* = 6.69, *p* < 0.01) and mental health (*t* = 4.94, *p* < 0.01). Higher score of social integration indicated higher physical health and mental health (*t* = 5.66, *p* < 0.01; *t* = 10.02, *p* < 0.01). And higher score of social trust also represented a higher physical health (*t* = 3.27, *p* < 0.01) and mental health (*t* = 5.51, *p* < 0.01) as well. The young migrant older adults aged from 60 to 69 years had a better physical health and mental health than another group aged above 70 years (*t* = 2.05, *p* = 0.04; *t* = 2.54, *p* = 0.01). Migrant older adults in marriage had a higher physical health (*t* = 3.16, *p* < 0.01) and mental health (*t* = 3.04, *p* < 0.01) than those who were not-in-marriage. Compared with female migrant older adults, the male both in urban-to-urban and rural-to-urban groups had a better physical health (*t* = 2.49, *p* = 0.01). Years living in Hangzhou did not demonstrated a statistically influencing on physical health and mental health.

**Table 6:** Summary of multiple liner regression models on physical health and mental health

Models	Physical health					Models	Mental health				
	b	t	*p*	95% CI for b			b	t	*p*	95% CI for b	
				Lower	Upper					Lower	Upper
Model PH1 (R = 0.079, Adjusted R^2^ = < 0.015)						Model MH1 (R = 0.089, Adjusted R^2^ = < 0.017)					
Constant	294.07	73.82	< 0.01	286.25	301.89	Constant	289.06	75.46	< 0.01	281.54	296.58
Migrant status (rural-to-urban)	12.14	2.26	0.02	1.61	22.67	Migrant status (rural-to-urban)	12.93	2.50	0.01	2.77	23.08
Model PH2 (R = 0.481, Adjusted R^2^ = 0.227)						Model MH2 (R = 0.567, Adjusted R^2^ = 0.319)					
Constant	79.58	4.38	< 0.01	43.88	115.28	Constant	21.28	1.32	0.19	−10.37	52.93
Migrant status (rural-to-urban)	1.96	0.41	0.69	−7.52	11.43	Migrant status (rural-to-urban)	2.70	0.62	0.54	−5.90	11.30
Score of reciprocity	4.65	6.56	< 0.01	3.26	6.05	Score of social integration	3.98	10.41	< 0.01	3.23	4.74
Score of social integration	2.20	5.16	< 0.01	1.36	3.04	Score of social trust	3.41	5.51	< 0.01	2.20	4.63
Marriage status (not-in-marriage)	28.07	4.48	< 0.01	15.76	40.38	Score of reciprocity	3.50	5.46	< 0.01	2.24	4.75
Score of social trust	2.41	3.54	< 0.01	1.07	3.74						
Model PH3 (R = 0.511, Adjusted R^2^ = 0.247)						Model MH3 (R = 0.599, Adjusted R^2^ = 0.348)					
Constant	69.03	3.13	< 0.01	25.75	112.31	Constant	28.21	1.47	0.14	−9.39	65.80
Migrant status (rural-to-urban)	−3.23	−0.58	0.56	−14.15	7.69	Migrant status (rural-to-urban)	0.51	0.11	0.91	−8.82	9.84
Score of reciprocity	4.71	6.69	< 0.01	3.33	6.09	Score of social integration	3.84	10.02	< 0.01	3.09	4.59
Score of social integration	2.46	5.66	< 0.01	1.61	3.31	Score of social trust	3.18	5.15	< 0.01	1.97	4.39
Marriage status (not-in-marriage)	20.50	3.16	< 0.01	7.78	33.21	Score of reciprocity	3.12	4.94	< 0.01	1.88	4.36
Score of social trust	2.24	3.27	< 0.01	0.90	3.58	Age (70 years and above)	14.82	2.54	0.01	3.35	26.28
Age (70 years and above)	13.75	2.05	0.04	0.56	26.95	Marriage status (not-in-marriage)	17.38	3.04	< 0.01	6.15	28.62
Gender (female)	12.44	2.49	0.01	2.63	22.24	Years living in local city(6 months to 1 year)					
Mainly economic source (offspring)	12.53	2.27	0.02	1.70	23.36	*One to three years*	−17.50	− 1.88	0.06	−35.77	0.78
Years living in local city(6 months to 1 year)						*Three to six years*	−12.54	− 1.42	0.16	− 29.90	4.83
*One to three years*	−10.92	−1.05	0.29	−31.34	9.49	*six to twenty years*	−20.49	−2.26	0.02	−38.31	−2.67
*Three to six years*	−12.16	− 1.23	0.22	−31.54	7.23	Weight status (Normal)					
*six to twenty years*	−18.75	− 1.85	0.07	−38.69	1.19	*Low weight*	−10.17	−0.87	0.38	−33.13	12.79
Weight status (Normal)						*Overweight*	8.31	1.83	0.07	−0.60	17.22
*Low weight*	−22.06	−1.70	0.09	−47.53	3.41	*Obesity*	0.03	< 0.01	1.00	−19.90	19.96
*Overweight*	7.34	1.43	0.15	−2.72	17.39	Educational attainment (Junior school and above)	−7.82	−1.61	0.11	−17.36	1.71
*Obesity*	−10.43	−0.94	0.35	−32.15	11.28						
Educational attainment (Junior school and above)	− 0.89	− 0.16	0.87	−11.54	9.76						

## Discussion

The urban-to-urban and rural-to-urban migrant older adults indeed had a vital difference across age, educational attainment, and the main source of livelihood. For example, the majority of urban-to-urban migrant older adults (85.58%) were self-supporting or dependent on their spouse, while that was only 51.11% in rural-to-urban migrant older adults. A higher proportion of urban-to-urban migrant older adults had a junior school and above (76.85%), while only 32.14% in rural-to-urban migrant older adults. Briefly, comparing with rural-to-urban migrant older adults, more urban-to-urban migrant older adults were more educated and economically independent.

In general, the score of physical health was higher in urban-to-urban migrant older adults than that in rural-to-urban migrant older adults, specifically for the dimension of Role Physical (RP) and Bodily Pain (BP). While these differences were disappeared after controlling variables such as gender, age, marital status, mainly economic source, cognitive social capital, and social integration. As a matter of fact, this demonstrated that there were no significant difference in physical health and mental health between urban-to-urban and rural-to-urban groups. Seemingly, this phenomenon can be explained by the theory of “health choice” (only *those older adults with a better health were more likely to flow, on the contrary, those with poor health had a lower possibility of mobility*). Actually, the majority of the urban-to-urban and rural-to-urban migrant older adults were drawing to city for taking care of their children or grandchildren, the majority of them were health just as they reported, which was consistent with previous finding that the majority of migrant older adults had a positive self-report health [26].

There was a positive correlation between cognitive social capital (social trust and reciprocity) and physical health, and cognitive social capital and mental health, which was consistent with a previous research in Bogota, Colombia [27, 28], Italy [29], and Catalonia [30]. It is widely recognized that social integration had powerful effects on physical health and mental health in community members [31, 32]. Similarly, a positive linkage was observed between social integration and physical health, and social integration and mental health in migrant older adults. Neither the differences of physical health nor the differences of mental health between urban-to-urban and rural-to-urban migrant older adults were obtained. It is noticeable, however, that urban-to-urban group had a higher reciprocity and social integration than that in rural-to-urban group. That indicated, benefiting from higher cognitive social capital and social integration, that urban-to-urban migrant older adults had a better social adaptability than rural-to-urban migrant older adults. A good social adaptability could be thought as a protective factor for physical health and mental health among migrant older adults.

In addition, demographic factors such as marital status and age were vital factors for physical health and mental health among migrant older adults. Migrant older adults in marriage had better physical health and mental health than those who were not-in-marriage. Migrant older adults aged 60 to 69 years showed better physical health and mental health than others. Compared with female, male migrant older adults had a better physical health than female, while no significant difference was observed in mental health status for these two groups. That indicated, to achieve the goals of health policies for all, making health policies should focus not only on migrant older adults, but also on the most vulnerable groups, such as the female, widowed, and the oldest migrant older adults,.

## Conclusions

In this study, social capital and social integration were positively related to the physical health and mental health among migrant older adults, which played important roles in fostering human health. Giving the female, widowed, and the oldest migrant older adults were more vulnerable compared with other groups among migrant older adults, more health policies should be applied for them.

### Limitations

There were certain limitations in this study. First, only cognitive social capital was analyzed in this study, which might have reduced the power of the findings. Secondly, this study adopted a cross-sectional survey, which was weak to make a causal relationships between health and migrant status. However, this study compared the physical health and mental health between urban-to-urban and rural-to-urban migrant older adults, which provided a new respective for the research on migrant older adults’ health and a new clue for further intervention to improve migrant population’s health.

## Data Availability

The data supporting this study is available from the corresponding author for reasonable request.

## Abbreviations

ANOVA: Univariate Analysis of Variance
BMI: Body Mass Index
BP: Bodily Pain
CNY: China Yuan
GH: General Health
MANOVA: Multivariate Analysis of Variance
MH: Mental Health
PF: Physical Function
PH: Physical Health
RE: Role Emotional
RP: Role Physical
SF: Social Function
SF-36: 36-item Short Form Health Survey
USD: USA Dollar
VT: Vitality

## References

[1] Tonelli M, Riella M (2014). Chronic kidney disease and the ageing population. Nephron Clin Pract.

[2] Gong CH, Kendig H, He X. Factors predicting health services use among older people in China: An analysis of the China Health and Retirement Longitudinal Study 2013. BMC Health Serv Res. [Journal Article, 2016-02-18. 2016;16(1):–63. 10.1186/s12913-016-1307-8 PMC4758158 26892677

[3] Xiaodong Y, Shujuan W, Yaru X, Jing L (2011). A clinical follow-up study on the risk of cerebral infarction in Chinese aging overweight and obese population. Obes Res Clin Pract.

[4] Commission TFPD (2017). China’s floating population development report 2017.

[5] Oshio T (2016). The association between individual-level social capital and health: cross-sectional, prospective cohort and fixed-effects models. J Epidemiol Community Health.

[6] Lin Y, Zhang Q, Chen W, Shi J, Han S, Song X (2016). Association between Social Integration and Health among Internal Migrants in ZhongShan, China. PLoS One.

[7] Harpham T, Grant E, Thomas E (2002). Measuring social capital within health surveys: key issues. Health Policy Plan.

[8] Raymond-Flesch M, Auerswald C, McGlone L, Comfort M, Minnis A (2017). Building social capital to promote adolescent wellbeing: a qualitative study with teens in a Latino agricultural community. BMC Public Health.

[9] Vyncke V, De Clercq B, Stevens V, Costongs C, Barbareschi G, Jonsson SH (2013). Does neighbourhood social capital aid in levelling the social gradient in the health and well-being of children and adolescents? A literature review. BMC Public Health.

[10] Portes A (1998). Social capital: its origins and applications in modern sociology. Annu Rev Sociol.

[11] Woolcock M, Narayan D (2000). Social Capital. World Bank Res Obser.

[12] Harpham T, Grant E, Rodriguez C (2004). Mental health and social capital in Cali, Colombia. Soc Sci Med.

[13] Kawachi I. Social capital and community effects on population and individual health. Ann N Y Acad Sci. [Journal Article; Review]. 1999-01-19;896:120–130. 10.1111/j.1749-6632.1999.tb08110.x 10681893

[14] Cannuscio C, Block J, Kawachi I. Social capital and successful aging: the role of senior housing. Ann Intern Med. [Journal Article; Review]. 2003-09-02;139(5 Pt 2):395–399. 10.7326/0003-4819-139-5_part_2-200309021-00003 12965964

[15] Villalonga-Olives E, Kawachi I. The measurement of bridging social capital in population health research. Health Place. [Journal Article; Review].2015-11-01;36:47–56. 10.1016/j.healthplace.2015.09.002 26409896

[16] Leedahl SN, Chapin RK, Little TD. Multilevel examination of facility characteristics, social integration, and health for older adults living in nursing homes. J Gerontol B Psychol Sci Soc Sci. [Journal Article; Research Support, Non-U.S. Gov't]. 2015-01-01;70(1):111–122. 10.1093/geronb/gbu112 25213304

[17] De Silva MJ, Huttly SR, Harpham T, Kenward MG. Social capital and mental health: a comparative analysis of four low income countries. Soc Sci Med. [Journal Article; Research Support, Non-U.S. Gov't]. 2007-01-01;64(1):5–20. 10.1016/j.socscimed.2006.08.044 17045716

[18] Loret DMC, Stanojevic S, Ruiz P, Gilman RH, Smeeth L, Miranda JJ. The effect of rural-to-urban migration on social capital and common mental disorders: PERU MIGRANT study. Soc Psychiatry Psychiatr Epidemiol. [Journal Article; Research Support, N.I.H., Extramural; Research Support, Non-U.S. Gov't]. 2012-06-01;47(6):967–73. 10.1007/s00127-011-0404-6 PMC3248919 21667301

[19] Ramlagan S, Peltzer K, Phaswana-Mafuya N. Social capital and health among older adults in South Africa. BMC Geriatr. [Journal Article; Research Support, N.I.H., Extramural; Research Support, Non-U.S. Gov't]. 2013-09-28;13:100. 10.1186/1471-2318-13-100 PMC3851859 24073666

[20] Statistical bulletin of the national economic and social development of Hangzhou in 2012.; 2013.

[21] Jingcheng G. A survey report of rural to urban population's social integration in Hangzhou. City Monitor. 2014 2014-04-20(02):91–103.

[22] Frank C, Davis CG, Elgar FJ. Financial strain, social capital, and perceived health during economic recession: a longitudinal survey in rural Canada. Anxiety Stress Coping. [Journal Article; Research Support, Non-U.S. Gov't]. 2014 2014-01-20;27(4):422–438. 10.1080/10615806.2013.864389 24251877

[23] Li S, Delva J. Social capital and smoking among Asian American men: an exploratory study. Am J Public Health. [Journal Article; Research Support, N.I.H., Extramural; Research Support, U.S. Gov't, P.H.S.]. 2012 2012-05-01;102 Suppl 2:S212–S221. 10.2105/AJPH.2011.300442 PMC3359821 22401511

[24] Yuan R, Nan Q. Social integration for migrants: process, measurement and determinants. Popul Res. 2010 2010-03-29(]):11–20.

[25] Li L, Wang H, Shen Y. Development and psychometric tests of a Chinese version of the SF-36 Health Survey Scales. Zhonghua Yu Fang Yi Xue Za Zhi. [Journal Article; Validation Studies]. 2002 2002-03-01;36(2):109–113. 12410965

[26] Jing G, Liping X, Hui F (2017). Status and influencing factors of self-rated health among floating elderly population: an analysis with ordinal logistic regression. Chin J Public Health.

[27] Peigang W, Xin'Guang C. Social capital, social integration, and acquisition of health: taking rural to urban migrant population as an example. J Huazhong Univ Sci Technol (social science edition). 2015 2015-05-10(03):81–88.

[28] Lucumi DI, Gomez LF, Brownson RC, Parra DC. Social capital, socioeconomic status, and health-related quality of life among older adults in Bogota (Colombia). J Aging Health. [Journal Article; Research Support, Non-U.S. Gov't]. 2015 2015-06-01;27(4):730–750. 10.1177/0898264314556616 PMC4755298 25370712

[29] Fiorillo D, Sabatini F (2015). Structural social capital and health in Italy. Econ Hum Biol.

[30] Stoyanova A, Diaz-Serrano L (2009). Differential impact of social capital on mental health in the native-born and immigrant populations living in Catalonia (Spain). Gac Sanit.

[31] Gouin JP, Zhou B, Fitzpatrick S (2015). Social integration prospectively predicts changes in heart rate variability among individuals undergoing migration stress. Ann Behav Med.

[32] Orwelius L, Backman C, Fredrikson M, Simonsson E, Nordlund P, Samuelsson A (2011). Social integration: an important factor for health-related quality of life after critical illness. Intensive Care Med.

